# Reduced Psychosocial Well-Being among the Children of Women with Early-Onset Breast Cancer

**DOI:** 10.3390/curroncol30120731

**Published:** 2023-11-21

**Authors:** Antje Schliemann, Alica Teroerde, Bjoern Beurer, Friederike Hammersen, Dorothea Fischer, Alexander Katalinic, Louisa Labohm, Angelika M. Strobel, Annika Waldmann

**Affiliations:** 1 Institute for Social Medicine and Epidemiology, University of Luebeck, 23562 Luebeck, Germanyalexander.katalinic@uksh.de (A.K.); louisa.labohm@uksh.de (L.L.); 2 Department of Obstetrics and Gynaecology, Ernst von Bergmann Clinic, 14467 Potsdam, Germany; 3 Department of Obstetrics and Gynaecology, University Hospital of Schleswig-Holstein, Campus Luebeck, 23562 Luebeck, Germany

**Keywords:** early-onset breast cancer, maternal health, maternal well-being, child health, child well-being, strengths and difficulties questionnaire (SDQ)

## Abstract

Background: Approximately 27% of female breast cancer patients are diagnosed before the age of 55, a group often comprising mothers with young children. Maternal psychosocial well-being significantly impacts these children’s psychosocial well-being. This study assesses the well-being of children with mothers who have early-onset breast cancer. Methods: We examined the eldest child (up to 15 years old) of women with nonmetastatic breast cancer (<55 years old, mean age: 40) enrolled in the mother–child rehab program ‘get well together’. Using maternal reports on children’s well-being (the Strengths and Difficulties Questionnaire; SDQ), we describe the prevalence of abnormally high SDQ scores and identify protective and risk factors via linear regression. Results: The mean SDQ scores of 496 children (4–15 years old, mean age: 8) fell below the thresholds, indicating psychosocial deficits. However, most SDQ scores deviated negatively from the general population, especially for emotional problems, with one in ten children displaying high and one in five displaying very high deficits. Female sex, more siblings, a positive family environment and maternal psychosocial well-being were protective factors for children’s psychosocial well-being. Conclusions: Children of mothers with breast cancer may benefit from improved maternal well-being and family support. Further research is needed to identify appropriate interventions.

## 1. Introduction

Children’s psychosocial well-being is a fragile construct that is influenced, among other factors, by parental health [1]. The existing literature addresses the psychosocial impact of serious parental illness on children. However, the results are conflicting, as they demonstrate neither a purely positive nor a purely negative effect [2].

The diagnosis of (breast) cancer is a life-changing experience for every individual and brings many new challenges [3]. Among women diagnosed with breast cancer, three out of ten are 54 years old or younger at the time of diagnosis [4]. These young women often face a double burden: on one hand, they are deeply rooted in family structures, being the cornerstone of the family, and on the other hand, they follow a professional career [5]. A diagnosis of breast cancer initially disrupts their lives as they may not be able to continue working and may struggle to fulfil their roles within the family and society. Moreover, the disease can lead to both psychosocial and aesthetic changes that require intense coping [6].

This new situation cannot be hidden from the children, forcing them to face emotional challenges [3]. In addition to the physical and psychological changes in their mothers, children are affected by their mother’s absence due to various treatment sessions. This absence can cause anxiety, worry and feelings of neglect [7].

A study conducted by a team of researchers at the Philipps University in Marburg, Germany, in 2006 found that the psychosocial well-being of children of early-onset breast cancer patients was significantly impaired before and at the start of a 3-week inpatient mother–child rehab program called ‘get well together’. While most children benefitted from the rehab program, about 10% of the children still experienced emotional distress one year after participation in the program [8]. Although it is an important evaluation study, the Marburg study did not investigate the protective or risk factors that might influence the psychosocial well-being of children. The knowledge about these factors could be used to design proactive interventions and comprehensive support for affected children in the future.

The literature suggests that disclosure of the illness within the family and maintaining a stable family climate generally have a positive effect on children’s psychosocial well-being [2,9]. Social support, in the form of parents’ social environment, school interactions and external help, has been identified as a protective factor. Other factors influencing the psychosocial outcomes of children facing a parental cancer diagnosis seem to be the children’s age when confronted with the diagnosis as well as gender [2].

Given these circumstances, the question arises as to how young children (≤15 years old) whose mothers have been diagnosed with early-onset nonmetastatic breast cancer cope with the situation. We therefore analysed data from this specific group of children to answer the following research questions: What is the prevalence of psychosocially distressed children among children facing maternal breast cancer, stratified by sex and age? What are the most common areas of distress? To what extent are these children distressed compared to their age-matched controls from the general population or to at-risk groups? We also investigated how contextual factors related to the mother’s breast cancer diagnosis influence children’s psychosocial well-being. In general, we were interested in identifying which children are particularly affected or at risk and which risk and protective factors exist, allowing us to identify potential deficits early and provide support to affected children. Lastly, the timing of recruitment raised another research question: to what extent has the COVID-19 pandemic influenced coping and the psychosocial well-being of these children?

## 2. Materials and Methods

### 2.1. Study Population

Our prospective cohort study focused on a group of young children aged 4 to 15 years old whose mothers had been diagnosed with early-onset breast cancer and who took part in the ‘get well together’ rehab program at the Ostseedeich Clinic in Groemitz, Germany.

To be eligible for the 3-week inpatient mother–child rehab program, women had to have an initial diagnosis of nonmetastatic breast cancer (ICD-10 C50) or ductal carcinoma in situ (DCIS; ICD-10 D05). Adjuvant therapy (surgery, chemotherapy or radiotherapy) had to be completed at least six weeks before enrolment. Women who were diagnosed with distant metastases at the time of diagnosis were excluded from the rehab program. Further, they had to be accompanied by at least one child ≤15 years of age (before the time of the pandemic, i.e., before March 2020, the age limit for accompanying children was ≤12 years old). For the purpose of our study, women had to be under 55 years of age at the time of the breast cancer diagnosis.

For the present analysis, recruitment took place between January 2019 and July 2021. As a consequence of the onset of the COVID-19 pandemic, the rehab clinic was shut down by the end of March 2020 and reopened only in July 2020. As the COVID-19 pandemic has been shown to have a significant impact on the psychosocial well-being of both adults and children [10,11], we divided our study participants into two groups. One group consisted of children and mothers with a rehab stay before the pandemic (until March 2020; about 35–40 women per 3-week rehab program). The other group consisted of children and mothers who attended the rehab clinic during the COVID-19 pandemic (from July 2020 onward, 25 women per 3-week rehab program). This distinction allowed us to analyse the specific impact of the global spread of the virus on the mental health of an already burdened group of breast cancer patients and their children.

### 2.2. Data Sources

During the rehab stay, we asked women to complete a paper-based study questionnaire. To assess the psychosocial well-being of the oldest child accompanying each mother, we included the Goodman’s Strengths and Difficulties Questionnaire (SDQ) in our study questionnaire [12]. The SDQ is an internationally validated screening tool for the assessment of various behavioural aspects in children and adolescents [13]. The questionnaire includes items to analyse emotional problems, conduct problems, hyperactivity, peer problems and prosocial behaviour in children. The sum of the scores from the first four scales was calculated to give a total difficulties score (range of scores: 0–40). High scores on these scales and on the total difficulties score indicate a negative behavioural tendency in the child [12]. The prosocial behaviour scale was considered separately and was not included in the total difficulties score, as high scores on this scale indicate positive behaviour [12]. We used the cut-off values defined by Goodman et al. for the different scales of the SDQ (Supplemental Table S1). In order to contextualise the scores on the SDQ scales and to be able to evaluate conspicuous results, the data were compared to three clinical risk groups (paediatric outpatient, paediatric rehabilitation and paediatric psychiatry) as reported in Woerner et al. [14]. They were also compared with a norm sample of children aged six to sixteen years old [14] and children from the German general population [15]. In addition to the SDQ questions, general questions about the child’s age, sex and siblings were asked, as well as other questions about the family climate (a condensed version of Schneewind et al.’s Family Climate Test System [16]). The age of the children was divided into five developmental stages according to Erikson’s model of psychosocial development: babyhood 0–<1.5 years old, toddler age 1.5–3 years old, playing age 4–5 years old, school age 6–11 years old, adolescence 12–17 years old and young adults 18–39 years old (simplified representation) [17,18].

The study questionnaire included five validated instruments to assess maternal psychosocial well-being, covering domains such as quality of life (using the EORTC QLQ-C30, European Organisation for Research and Treatment of Cancer quality of life core questionnaire [19]), depression (PHQ-9, Patient Health Questionnaire [20,21]), distress (Herschbach et al.’s FBK questionnaire [22,23]) and social support (Dalgard et al.’s 3-item Oslo Social Support Scale [24]). After the women had completed the rehab program, clinical, tumour-specific and treatment information were extracted from the rehab record files. These included admission forms, rehab discharge letters and previous medical reports and findings from hospital stays.

### 2.3. Statistics

The original analysis plan included several measures of maternal psychosocial well-being as explanatory variables for the children’s SDQ total difficulties scores. Correlation analyses (using Spearman correlation) revealed a high correlation between these variables. Therefore, an exploratory factor analysis (principal component analysis with oblique rotation) was conducted to determine whether these variables represented a common factor. The results showed that the variables burden (FBK), depression (PHQ-9), fatigue, social function, role function, physical function, emotional function and global quality of life/global health status (all from QLQ-C30) loaded on a common factor. With an eigenvalue of 4.90, 61.2% of the variance was explained. This common factor was subsequently termed maternal ‘psychosocial well-being’. It was presented on a scale that was realised through mean aggregation (at least five out of seven variables needed to have valid values) after transforming all the variables/scores into a range from 0 to 100 and uniform polarisation (higher values corresponding to a higher quality of life, higher functionality, lower psychological distress and lower levels of fatigue).

**Figure 1:**
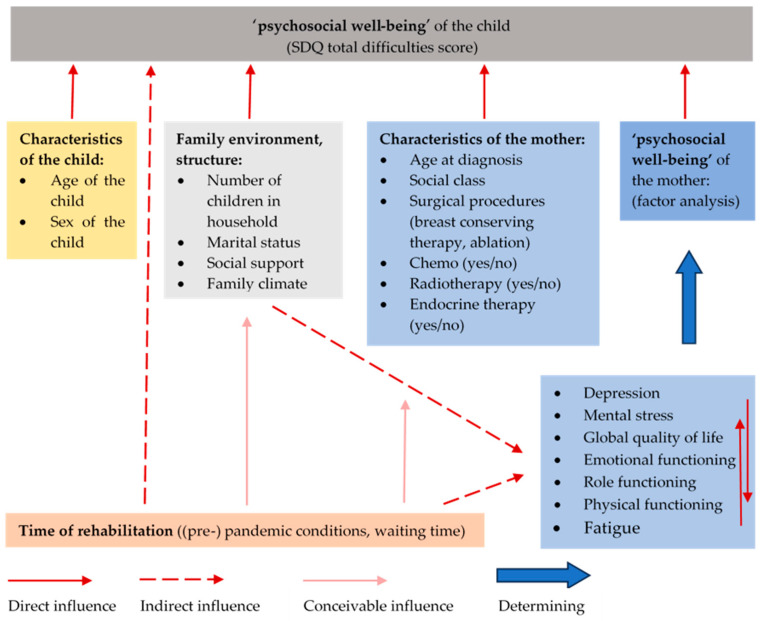
Overview of possible influencing factors on the SDQ total difficulties score.

To identify risk and protective factors for children’s psychosocial well-being, a multivariable linear regression analysis was performed with the outcome variable ‘total difficulties score’. An overview of potential factors influencing children’s well-being is shown in Figure 1. The variables ‘recruitment during the pandemic yes/no’, age and sex of the child, information on family structure (social support, family climate, number of children in the household, social class and single parenting), as well as information on tumour therapy (age at diagnosis, chemotherapy, radiotherapy and endocrine therapy) and the factor ‘psychosocial well-being’ of the mother were included as predictors in the multivariable linear regression model in blocks one after the other (method: blockwise inclusion).

Descriptive statistics were used to describe the sample, including means and measures of dispersion and absolute and relative frequencies. Differences between groups were analysed by using various statistical tests (a chi-squared test, Mann–Whitney U test, independent samples *t*-test and Kruskal–Wallis test). A *p*-value <0.05 was considered statistically significant.

Statistical analysis of the pseudonymised dataset was performed by using SPSS version 22 [25], and graphs were generated by using the statistical program R version 4.1.0 [26] with the ggplot2 package [27].

### 2.4. Ethics

The prospective cohort study was conducted in accordance with the Declaration of Helsinki. The study protocol was approved by the ethics committee of the University of Luebeck (Ref. No. 10-096; amendment 7 August 2018). All participants gave written informed consent to participate in this study.

## 3. Results

The present study examines a group of 496 children and their mothers diagnosed with early-onset breast cancer. By the start of the COVID-19 pandemic, at the end of March 2020, 253 women and children were enrolled in this study. The remaining 243 women and children participated in the rehab program from July 2020 to July 2021, after the COVID-related shut down.

### 3.1. Characteristics of the Mothers

The demographic and clinical characteristics of the mothers/breast cancer patients are shown in Table 1. The mean age at diagnosis was 40 years old, with the prepandemic group being younger than the COVID-19 pandemic group on average. More than 80% of the women had an early-stage tumour (Tis, T0 or T1). About 27% showed lymph node involvement. Poorly differentiated tumour cells (grade G3) were found in just over half of the cases. Notably, the G3 stage occurred in almost 10% more women from the prepandemic period than from the pandemic period (prepandemic group = 60.1% vs. pandemic group = 49.1%, *p* = 0.031).

All women in this study underwent surgery: 57.5% underwent breast-conserving surgery, 11.5% chose radical mastectomy without reconstruction and 31.0% had breast reconstruction. Chemotherapy was given to 85.9% of women, radiotherapy to 81.7%, antibody therapy to 25.8% and endocrine therapy to 71.2%. A statistically significant difference was observed for the waiting time between the start of therapy and the start of rehabilitation (i.e., the date the questionnaire was completed during the rehab stay). This is most likely due to the shut down of the rehab clinic between March and July and the reduced number of women per 3-week rehab program from July 2020 onward.

About two-thirds of the women reported being in a partnership or being married, while about 15% were separated or divorced. The women surveyed had an average of 1.83 children, and almost 85% of them had a maximum of two children (Table 2).

### 3.2. Characteristics of the Children

The demographic characteristics of the children are presented in Table 3. The analysis of the children focused on the oldest child who accompanied the mother to the rehab facility. The average age of these children was 8.6 years old (SD 2.8) and the range was 4 to 15 years old. The majority of these children (67.5%) were of school age at the time of rehab. Of the children studied, 53.5% were girls. Approximately 35% of the children were single children.

### 3.3. Psychosocial Well-Being in Children

**Figure 2:**
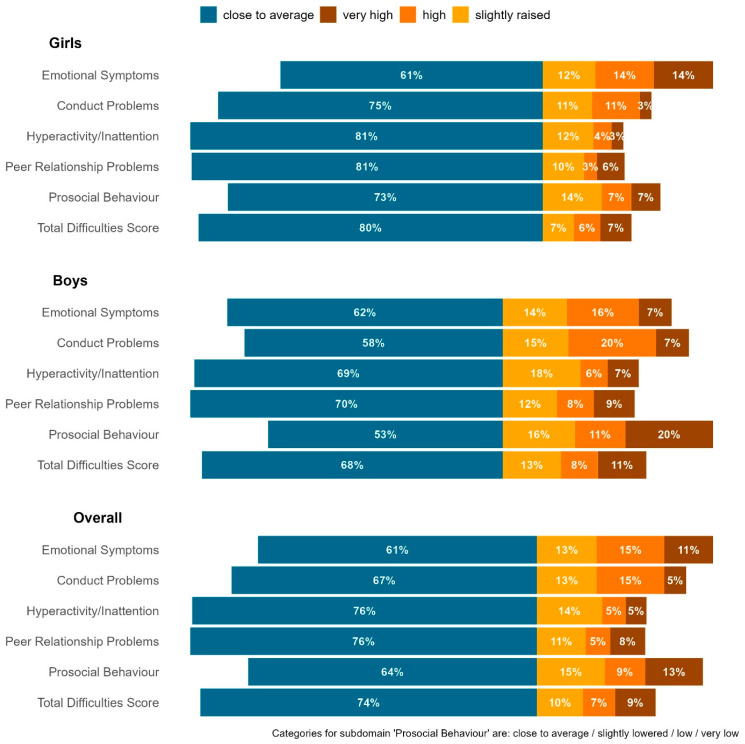
Maternal assessment of eldest child (frequency distribution of each category on the SDQ scales).

The mean SDQ total difficulties score was 10.4 points with a standard deviation of 5.9 points, indicating that, on average, the children had inconspicuous/normal scores. But, approximately 26% of the children had borderline to significantly abnormal SDQ total difficulties scores (scores within the rage of 17 to 40 points), indicating psychosocial distress (see Figure 2: overall). This group further differentiates into 9% of the children with very high scores, 7% with high scores and 10% with slightly raised scores (Figure 2). In total, 22% of the children showed significant to very significant deficits in the areas of prosocial behaviour and emotional problems. A further 20% of children had high to very high conduct problems.

When the data were stratified by sex, clear differences emerged. Boys showed higher deficits in all categories except emotional problems. This difference was most pronounced in the area of prosocial behaviour, where boys had twice as many deficits (31% ranging from high to very high) as girls (14% ranging from high to very high). In the category of emotional problems, girls had twice as many emotional deficits (14% in the very high range) compared to boys (7% in the very high range).

The analysis of sex differences showed significant differences to the detriment of boys in the categories of conduct problems (*p* < 0.001), hyperactivity/inattention (*p* < 0.001), prosocial behaviour (*p* < 0.001) and total difficulties score (*p* < 0.008).

**Figure 3:**
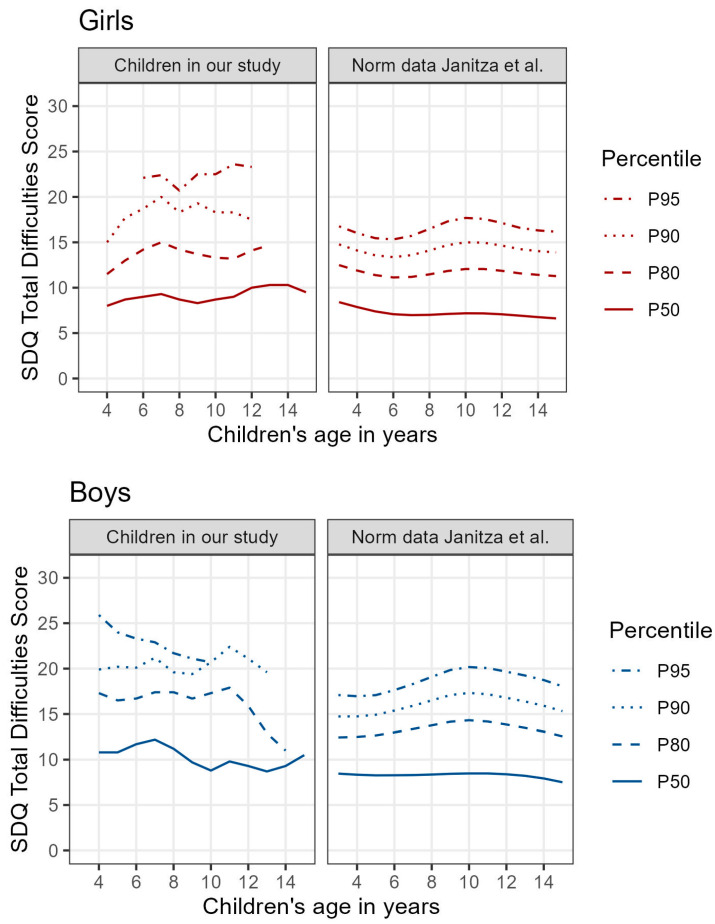
SDQ total difficulties score: differences in stress depending on sex and age.

In our study, there is a clear sex difference in the progression of the percentile curves over the age of children (Figure 3). While girls showed increasing total difficulties scores with increasing age, boys showed high scores in their younger years that decreased with increasing age. This effect is not found in the normal population of the same age by Janitza et al. [15]. In our study cohort, 10% of the boys and 5% of the girls of all ages had scores above 20 points on the total difficulties score, meaning they were very severely and more distressed than the comparison group.

**Figure 4:**
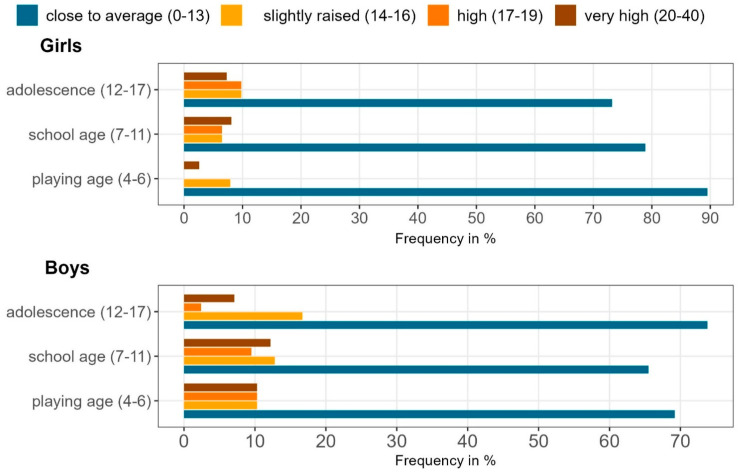
SDQ total difficulties score: differences in distress depending on sex and age.

When categorising age according to Erikson’s stages (Figure 4), more than a quarter of both school-aged children and adolescents showed abnormal total difficulties scores indicating a reduced psychosocial well-being. At school age, 34.5% of the boys were affected, compared with 21.1% of the girls (*p* = 0.049). At playing age, 30.8% of the boys and 10.5% of the girls were affected. In older children (adolescents), the sex differences became less pronounced, with boys and girls showing similar levels of improved psychosocial well-being (boys = 26.2% vs. girls = 26.8%, *p* = 0.453).

In our study, boys were particularly vulnerable at younger ages while girls showed increasing distress with increasing age, but not significantly more than boys at any developmental stage.

### 3.4. Comparison of SDQ Scores with Normative and Other Clinical Cohorts

The mean SDQ total difficulties score in our study (10.4 points) was higher than that of Woerner et al. (8.13 points) but lower than that of the paediatric outpatient clinic (8.18 points) (Table 4). In contrast, the values of the children in our study were significantly lower than those in paediatric rehabilitation (16.8 points) and paediatric psychiatry (16.2 points). Using the threshold of 16 points, approximately 19% of the children in our study were classified as distressed. This was almost double the proportion of distressed children in the normative sample but less than half the proportion of children in rehabilitation or psychiatric treatment [14].

With regard to emotional problems, the mean value for the children in our study (3.08 points) exceeded the mean value of the cohort of Woerner et al. (1.53 points) and of the paediatric outpatient clinic (2.14 points). Compared with children in a paediatric rehabilitation (4.19 points), the children in our study had better scores and approached the paediatric psychiatry data (3.67 points). Most of the scores were below the cut-off of four points, suggesting that most of the children in our study were not distressed. However, the frequency of (very) high scores (defined as ≥5 points) among them was about 3.5 times higher than in the general population.

In terms of conduct problems and hyperactivity, the children in our study had higher mean scores (2.04 points and 3.77 points) than the children in the cohort of Woerner et al. (1.82 points and 3.19 points) and in the paediatric outpatient clinic (1.79 points and 3.02 points). In contrast, they had better scores than those in paediatric rehabilitation (3.51 points and 5.42 points) and the psychiatric facility (3.59 points and 5.72 points).

The comparison of the category peer problems showed similar results. With a mean of 1.49 points, the score of the children in our study was slightly below that of the cohort of Woerner et al. (1.59 points) and slightly above that of the paediatric outpatient clinic cohort (1.23 points), but significantly below that of the paediatric rehabilitation (3.66 points) and paediatric psychiatry group (3.17 points).

Regarding prosocial behaviour, the mean score of the children in our study (7.86 points) was higher than in the groups of Woerner et al. (7.55 points), the paediatric rehabilitation (7.39 points) and the paediatric psychiatry (6.69 points). This suggests better prosocial behaviour in children in our study. However, the children in our study showed lower values than those in the paediatric outpatient clinic (8.23 points). Taken together, the children in our study showed borderline abnormalities but were hardly distinguishable from the comparison group and outperformed the predistressed children.

### 3.5. Factors Influencing Children’s Psychosocial Well-Being—Results of Multivariable Regression Analysis

The overall multivariable linear regression model accounted for 15.4% of the variance in the dependent variable (the SDQ total difficulties score). Significant predictors of the total difficulties score were identified, including the maternal psychosocial well-being factor, family climate, the child’s sex as well as the number of children in the family (Table 5).

## 4. Discussion

Overall, our results are encouraging to start with: despite the low cut-offs for distress, the majority of children with maternal breast cancer who are taking part in a mother–child rehab program are classified as psychosocially non-stressed. The proportion of children with normal scores ranged from 61% (scale of emotional problems) to 76% (scale of hyperactivity and peer problems) and amounted to 74% for the SDQ total difficulties score. However, around 10% of the children in our study were found to be highly distressed in terms of the SDQ total difficulties score. Every fourth to fifth child showed significant distress in areas such as emotional problems (around 26%), prosocial behaviour (around 22%) and conduct problems (around 20%).

Similar observations were made in an earlier study conducted by a research group in Marburg, which used data from the same rehab program. The Marburg research team found that the proportion of children with abnormal scores before rehab was 23.5%, and in our study, this value was 26% during rehab [8].

In our study, we found that sex, number of siblings, family climate and the psychosocial well-being of the mother had a significant effect on the SDQ total difficulties score. Female sex, an increasing number of siblings, a positive family climate and a good psychosocial well-being of the mother can serve as protective factors for the psychosocial well-being of children at risk. The impact of the pandemic on children’s psychosocial well-being, which was formulated as a secondary research question, proved to be insignificant in our specific study sample in contrast to other research [11].

### 4.1. Psychosocial Well-Being of Mothers with Early-Onset Breast Cancer

The psychosocial well-being of mothers with breast cancer can be influenced by various factors, such as external factors (disease, treatment and side effects), personal resources (age, stress, functionality and social class), family and social structures and the time of diagnosis and treatment. All these considerations were taken into account in this study, combining the psychosocial variables (quality of life, depression, stress and social support) into a common construct: maternal psychosocial well-being.

Studies have shown that female gender and young age at diagnosis are risk factors for psychosocial well-being [28,29,30,31,32,33]. It is also known that breast cancer patients are more susceptible to psychological distress than other cancer patients [34,35,36]. As described in the literature [37,38,39], the women in our study, who have a comparatively young age at onset, are affected by tumours with an unfavourable tumour biology and receive predominantly aggressive therapies. Even though all the women had nonmetastatic breast cancer, over 85% of the women received chemotherapy. Almost 30% had lymph node involvement. Almost a quarter of the tumours were triple negative and more than 50% were poorly differentiated (G3). These observations are in line with a retrospective study reported by Radecka et al. from the UK involving 2956 young women (less than 40 years old at diagnosis). Of these, almost 60% of the women had a G3-differentiated tumour and around 20% had a triple-negative tumour. The lymph node involvement in the British study was about 50% and hence higher than in the present study [40]. This may be due to the exclusion of women with distant metastases who often also have increased lymph node involvement.

In terms of family climate and social support, only minor deficits were identified in our study, and the majority of women reported having social support and good family structures. Hammersen et al. identified social support as an important positive influencial factor on the psychosocial needs of early-onset breast cancer patients [41]. The participants of our study consisted mainly of women from upper social classes. Studies show that a high level of education, good social support and physical functionality have a protective effect on the psychosocial well-being of people with illnesses [30,32]. This makes it all the more alarming that the current population is heavily burdened nevertheless. For example, it is striking that the women in our study report worse scores compared to the general population for all quality of life subdomains (except the global health status/quality of life score from the EORTC QLQ-C30). The differences are particularly large (up to 20 points) for cognitive and social functioning and for symptoms of tiredness, insomnia and shortness of breath. Financial difficulties also seem to affect the women in our study more than women in the general population. Only recently we reported that a high proportion of women with early-onset breast cancer had a need for supportive care based on the cut-offs for the EORTC QLQ-C30, which have been proposed by Lidington et al. [41,42].

### 4.2. Children’s Psychosocial Well-Being and Comparisons with the Woerner et al. Cohorts

In a direct comparison, the ‘children of women with early-onset breast cancer’ from the study at hand show no distress on average in terms of the SDQ total difficulties score and only half the rate of distress compared to the risk groups from paediatric rehabilitation or psychiatry. However, they still show worse outcomes than children from the normative sample or from paediatric clinics [14]. On the emotional problems scale, the children show, on average, a similar level of distress as children from psychiatric institutions, with the distress twice as high as in the normative sample. Although the average scores of our children are not indicative of distress, severe or very severe distress is still almost four times more common in our cohort than in the normative sample. These levels suggest that maternal breast cancer and the consequences of the diagnosis and treatment on daily life have a significant emotional impact on some children and that they are not adequately supported, emphasizing the urgent need for intervention. This suggestion is consistent with a comprehensive study by Zheng et al. [43], which reported that a parental cancer diagnosis has a negative impact on children’s psychosocial well-being. Therefore, it was expected that a maternal breast cancer diagnosis would impact the SDQ scores of the children in our study. Regarding peer problems, there are no significant differences between the children in our study and the normative sample of Woerner et al. [14], but there are differences in the described risk groups, where such problems are more prevalent. This suggests that maternal illness, unlike illness in the children themselves, does not seem to affect children’s peer relationships. In terms of prosocial behaviour, the children in our study show, on average, borderline distress. However, their scores are slightly better than Woerner et al.’s normative group and significantly better than the preaffected children from clinical cohorts. This could be attributed to the fact that children learn to be more considerate, empathetic and appreciative of others through their mother’s illness. This hypothesis is supported by a study conducted by Inhestern et al. with 78 cancer patients, the majority of whom had been diagnosed with breast cancer. Nearly 38% of respondents reported that their children exhibited positive behaviours resulting from their illness. The children became more helpful, independent and responsible. They also stand out positively with improved academic performance and openness [44].

### 4.3. Factors Influencing Children’s Psychosocial Well-Being

While, in our study, the variable age of the child alone does not seem to have a significant impact on children’s psychosocial well-being, it is interesting that the combination of age and sex shows significant associations in school-aged children (according to Erickson). While in younger children, boys are almost three times more likely than girls to have abnormal scores on the SDQ total difficulties score, this association is not found in older children, and the sexes appear to be equally susceptible. Boys are particularly at risk at a young age, while girls are increasingly affected as they get older, but at no point in their development are they significantly more affected than boys (see Figure 3 and Figure 4).

Reiss et al. examine results from the years 2003–2008 of a nationwide cohort study, the BELLA study (‘survey on psychosocial well-being and behaviour’), which evaluates data from children and parents in the general population [45]. The authors focus on the relationship between socioeconomic status and children’s stressful life situations. They show that younger children in particular were more susceptible to stressful life situations, with boys recovering better than girls during a 2-year follow-up [45]. The COPSY study [46], which examined the psychosocial well-being of children and adolescents during the COVID-19 pandemic, showed that female gender and increasing age had a protective effect on psychosocial well-being when assessed with the SDQ. Only on the emotional problems scale do girls show more distress. On the peer problems scale, the risk increases with age and is independent of gender. This observation is surprising, as female gender is a risk factor for psychological distress in the adult population [35,47].

One explanation for the observed sex differences may lie in the close relationship between boys and their mothers, in which the Oedipus complex and similar aspects may play a role. At a young age, boys are generally very focused on their mothers, which may lead them to perceive them as much more vulnerable, especially when ‘mummy is sick’. Over time, this fixation weakens and a certain distance from the mother may develop. During puberty, boys generally share less with their mother and become more interested in other aspects of their life.

In contrast, girls may experience more stress during puberty. They identify more with their mother, their own bodies and therefore with possible illnesses. Girls tend to have less doubt about their health at this stage than boys. This may indicate that boys tend to retain childhood coping strategies for longer.

However, it is important to note that this hypothesis should be interpreted with caution as it is a simplistic representation of gender dynamics and the development of coping strategies. Further empirical research and analysis would be required to confirm or refute these hypotheses.

In our study, the pandemic had no effect on children’s psychosocial well-being. This differs significantly from the results of other studies [46,48,49] and suggests that the prepandemic questionnaire did not ask about important elements of the impact of the pandemic (e.g., school performance, exposure at home and school and lack of social contacts). Christian et al. [48] concluded from an online survey of 2672 children (3–10 years old) that stress levels were significantly higher during the pandemic. The authors identified single parenting and the absence of siblings as risk factors for emotional problems and a tendency to experience hyperactivity. This could also be a reason why the children in our sample showed comparable SDQ scores before and during the pandemic: the majority of mothers reported being in a stable partnership, and the majority of children had at least one sibling. Moreover, the study at hand showed the course of the pandemic until mid-2021 only. The long-term effects of the pandemic on children’s well-being should be part of future studies. There was also no association between the waiting time for taking part in the rehab program and SDQ scores. In fact, children whose mothers had short waiting times tended to be more stressed. In total, 25% of the children whose mothers had waited less than 6 months showed significantly increased SDQ total difficulties scores. Apart from the fact that the group of these children was very small and therefore not very representative (12 children), it can be assumed that a longer waiting time also implies a longer processing time [50]. Mothers and their children have the opportunity to better understand, accept and cope with the situation.

Chan et al. also found that an increasing number of siblings was associated with a lower prevalence of psychosocial distress in children, but female gender was a risk factor [50]. With regard to the number of children, it is important to bear in mind that mothers of single children may judge their children’s behaviour more harshly than mothers with several children, who may be used to a lot of activity.

While Christner et al. [48] were able to identify single parenthood as a risk factor for childhood stress, in line with the present study, a study by Weis et al. found no differences in SDQ scores between children of single and partnered mothers with cancer [51].

It is also noteworthy that maternal age at diagnosis did not seem to correlate with children’s psychosocial well-being. Finally, several studies have found an explicit relationship between the mother’s age at diagnosis and her psychological well-being [29,30,32], and the latter, as this study showed, was clearly correlated with the child’s psychosocial well-being. An indirect influence of the mother’s age on the child’s psychosocial well-being would therefore have been conceivable. In the present cohort, the mothers were all younger than 55 years old at the time of diagnosis and all had young children up to the age of 15. The participants were therefore very homogeneous in terms of life stage and the social and family role expectations of the women, so the age differences may not have been significant. It is also striking that the type of therapy and the stage of the mother’s disease did not have any effect on the SDQ total difficulties scores. In our study, women with distant metastases were excluded from rehab, so all the women in the group were in a potentially curative situation. It is possible that the group was too homogeneous in terms of disease to be able to make any conclusive assessment of the associations with the psychosocial outcome of the children. Alternatively, potential differences may only become significant when a different therapeutic approach (curative/palliative) is used.

### 4.4. Strengths and Weaknesses

A major strength of this study is the selection of the patient collective. It was a largely homogeneous group of mothers with early-onset nonmetastatic breast cancer. All the women were under 55 years of age, had no (distant) metastases at the time of diagnosis and were in oncological rehab with at least one of their children (≤15 years old). Because of the COVID-19 pandemic, the criteria for admitting children to the rehab clinic were changed, and the maximum age for children was raised from 12 to 15 years old, resulting in an overall age range of 4–15 years old, covering very different stages of development. This allowed for a detailed assessment of the influence of the childhood factor. A total of almost 500 women were enrolled in this study, so the data base was large enough to obtain valid results and also to allow for subgroup analyses. Data were collected by using established, standardised and validated questionnaires (e.g., SDQ, PHQ-9 and EORTC QLQ-C30). This allows for valid results and comparability with other studies. The fact that the groups of boys and girls and the prepandemic and pandemic groups were of similar size was a coincidence, but it is very gratifying, and it allowed the groups to be tested well statistically for differences or associations with other characteristics in an exploratory manner.

It should be noted, however, that the questionnaires used for the assessment of the children’s psychosocial well-being were completed by the mothers as part of an external assessment process; i.e., the children were not interviewed directly. This method of data collection required a comprehensive analysis of the sociodemographic and clinical characteristics and psychosocial well-being of the mothers. This was performed to mitigate potential biases in the assessment of the children’s psychosocial well-being, such as those arising from maternal depressive symptoms [52]. Further, the questionnaire was not amended after the onset of the pandemic with pandemic-related questions regarding the children. On the one hand, this might be the reason why we were not able to show an effect of the pandemic on psychosocial well-being. On the other hand, it is possible that the effect of the pandemic was there but was considerably smaller than the impact of the maternal breast cancer. And finally, data on race were not collected.

### 4.5. Implications for Clinical Practice

This study suggests that the psychosocial well-being of children from mothers with early-onset breast cancer is significantly affected. Currently, post-treatment and rehabilitation services for breast cancer patients focus primarily on the women themselves. The healthcare providers involved in post-treatment care should be aware that the psychosocial consequences of the disease may affect not only the women themselves but also their children. Maternal quality of life and a positive family climate have been identified as positive and modifiable factors influencing children’s psychosocial well-being. These factors should be monitored during post-treatment and rehabilitation, and interventions and support should be offered as needed to directly or indirectly improve children’s psychosocial well-being. An example of a successful intervention to improve the well-being of children is the study by Lewis et al. [53], which demonstrates the effectiveness of the ‘Enhancing Connections Program’ for female breast cancer patients with children aged 8–12 years old. The results suggest that such programs can help mitigate the psychosocial impact of maternal breast cancer on children. In particular, the program was shown to significantly improve mothers’ mood and parenting skills while positively influencing children’s behaviour and emotional adjustment in response to their mother’s breast cancer diagnosis. 

## 5. Conclusions

Children of women with nonmetastatic early-onset breast cancer show a reduced psychosocial well-being. Children’s well-being can be protected and improved by targeting maternal psychosocial well-being and family climate. Further research is needed to evaluate the effectiveness of these targeted interventions.

## Figures and Tables

**Table 1:** Characteristics of the mothers with early-onset breast cancer.

Characteristics	Statistics	Values
**Age of mother at diagnosis**	N	485
	Mean (SD *)	40.3 (5.34)
	Range	18–54
**Tumour size** (TNM T-category)	N	443
Tis	(in %)	8.4
T0		36.1
T1		36.3
T2		15.8
T3		3.4
T4		0
**Lymph node status** (TNM N-category)	N	425
N0	(in %)	72.9
N1		19.5
N2		6.1
N3		1.4
**Grading**	N	495
G1	(in %)	6.3
G2		39.1
G3		54.6
**Type of surgery**	N	496
Breast-conserving	(in %)	57.5
Mastectomy without reconstruction		11.5
Mastectomy/other with reconstruction		31.0
**Radiotherapy**	N	496
Yes	(in %)	81.7
No		18.3
**Chemotherapy**	N	496
Yes	(in %)	85.9
No		14.1
**Endocrine therapy**	N	496
Yes	(in %)	71.2
No		28.8
**Waiting time in months for the rehab program to start**	N	492
≤6 months	(in %)	4.1
7–12 months		39.2
13–18 months		49.8
>18 months		6.9
**Factor ‘psychosocial well-being’** (factor analysis)	N	488
	Mean (SD *)	61.1 (18.2)
	Range	18.3–98.5

* SD: standard deviation.

**Table 2:** Family environment and family structure.

Family Environment, Structure	Statistics	Values
**Number of Children ^#^**	N	495
1	(in %)	35.3
2		49.3
3		13.1
≥4		2.2
**Partnership**	N	494
Single	(in %)	18.4
Married		65.8
Living apart/divorced		14.8
Widowed		1.0
**Social Class Index** *(According to Deck and Roeckelein)*	N	494
Middle class	(in %)	35.0
Upper Class		65.0
**Social Support** *(3-item Oslo Social Support Scale)*	N	481
	Mean (SD *)	11.0 (1.86)
	Range	5–14
Little social support (3–8)	(in %)	8.9
Moderate social support (9–11)		50.1
Strong social support (12–14)		41.0
**Family climate**	N	491
	Mean (SD *)	68.1 (14.5)
	Range	19–96
Normal (>55.6–100)	(in %)	79.6
Small deficits (>48.2–55.6)		9.8
Strong deficits (0–48.2)		10.6

^#^ data refer to biological children where identifiable. * SD: standard deviation.

**Table 3:** Characteristics of the children.

Characteristics	Statistics	Values
**Age** **of the eldest child**	N	496
	Mean (SD *)	8.58 (2.78)
	Range	4–15
**Age categories by Erikson**	N	496
Playing age (4–5 years old)	(in %)	15.7
School age (6–11 years old)		67.5
Adolescence (12–17 years old)		16.7
**Sex**	N	493
Boys	(in %)	46.5
Girls		53.5
**Sibling status**	N	495
Single child	(in %)	35.4
At least one sibling		64.6

* SD: standard deviation.

**Table 4:** Comparison of children’s SDQ scores in our study with those of the Woerner et al. comparison groups [14].

	Children in Our Study	Norm Sample Woerner et al., 2002 [14]	Paediatric Outpatient Patients Hellweg, 2004, as Reported in Woerner et al. [14]	Paediatric Rehabilitation Patients Oepem et al., 2003, as Reported in Woerner et al. [14]	Paediatric Psychiatry Patients Becker et al., 2004, as Reported in Woerner et al. [14]
**Sample size**	*496*	930	995	1049	639
**Mean age in years**	*8.6*	10.7	9.9	11.4	10.5
**Proportion of boys (%)**	*46.5*	50.2	52.4	56.0	71.7
**Total difficulties score**					
Mean points	*10.4*	8.13	8.18	16.8	16.2
% ≥16	*18.8*	10.0	12.8	56.8	53.1
**Emotional problems**					
Mean points	*3.08*	1.53	2.14	4.19	3.67
% ≥5	*25.6*	7.7	14.1	45	35.8
**Conduct problems**					
Mean points	*2.04*	1.82	1.79	3.51	3.59
% ≥5	*10.1*	6.6	7.2	31.0	32.4
**Hyperactivity**					
Mean points	*3.77*	3.19	3.02	5.41	5.72
% ≥7	*15.3*	9.8	11.8	35.8	42.4
**Peer problems**					
Mean points	*1.49*	1.59	1.23	3.66	3.17
% ≥5	*7.7*	7.0	4.7	36.2	27.1
**Prosocial behaviour**					
Mean points	*7.86*	7.55	8.23	7.39	6.69
% ≥4	*6.0*	7.1	2.8	8.2	17.5

Italics = own data; normal font = literature/reference data.

**Table 5:** Results of the linear regression analysis with SDQ total difficulties score as the dependent variable.

	Non-Standardised Regression Coefficient Beta	*p*-Value
**Contextual factors**		
**Rehab stay before or during COVID-19 pandemic** (reference before versus during)	0.274	0.607
**Waiting time between diagnosis and rehab stay** (ref. <6, vs. 6–12 or >12 months)	−0.555	0.282
**Children’s characteristics**		
**Sex of the eldest child** (ref. male vs. female)	−1.63	0.002
**Age of the eldest child in years** (4–15)	0.008	0.937
**Descriptors of family life and social support**		
**Number of children in household** (1–5)	−0.786	0.031
**Single parenting** (ref. yes vs. no)	−0.720	0.266
**Family environment** (score range: 0–100; low values indicate problematic environment: the higher the better)	−0.109	<0.001
**Social support** (score range: 3–14; low values indicate low support: the higher the better)	−0.031	0.827
**Social status** (ref. middle vs. high)	−0.361	0.521
**Characteristics of the mothers with early-onset breast cancer**		
**Age in years at diagnosis**	−0.058	0.280
**Received systematic anticancer treatment (chemotherapy)** (ref. no vs. yes)	0.391	0.644
**Received radiotherapy** (ref. no vs. yes)	0.456	0.486
**Received endocrine therapy** (ref. no vs. yes)	0.543	0.337
**Psychosocial well-being** (score range: 0–100; low values indicate low psychosocial well-being: the higher the better)	−0.055	<0.001

## Data Availability

The dataset of this study is not publicly available because informed consent from the study participants did not cover the public deposition of data. The dataset is available from the corresponding author upon reasonable request.

## Supplementary Materials

The following supporting information can be downloaded at https://www.mdpi.com/article/10.3390/curroncol30120731/s1. Table S1 Categorization of the SDQ scores according to Woerner et al. and Goodman et al.
