# Supplementary material for: Reduced Psychosocial Well-Being among the Children of Women with Early-Onset Breast Cancer
Source: Curr Oncol. 2023 Nov 21;30(12):10057–74. doi: 10.3390/curroncol30120731 (PMC10742300; doi:10.3390/curroncol30120731)
Supplement: Supplementary file 1 [file curroncol-30-00731-s001.zip › curroncol-2665614-supplementary.pdf]

Supplemental Table S1 Categorization of the SDQ scores according to Woerner et al. and Goodman et al.

Scores	Classification according to Woerner et al. [14]*1			Classification according to Goodman et al. [12] *2			
**Original three-band categorisation**	Normal	Borderline (Cut-Off)	Abnormal	Normal	Borderline (Cut-Off)	Abnormal	
**Newer four-band categorisation**				Close to average	Slightly raised (/slightly lowered)	High (/Low)	Very high (very low)
**Total difficulties score**	0-12	13-15	16-40	0-13	14-16	17-19	20-40
**Emotional problems score**	0-3	4	5-10	0-3	4	5-6	7-10
**Conduct problems score**	0-3	4	5-10	0-2	3	4-5	6-10
**Hyperactivity score**	0-5	6	7-10	0-5	6-7	8	9-10
**Peer problems score**	0-3	4	5-10	0-2	3	4	5-10
**Prosocial behaviour score**	6-10	5	0-4	8-10	7	6	0-5

*1 SDQ validated for children aged 4-17 years

*2 SDQ validated for children aged 6-16 years
